# The metabolites of lactic acid bacteria: classification, biosynthesis and modulation of gut microbiota

**DOI:** 10.15698/mic2023.03.792

**Published:** 2023-02-08

**Authors:** Huang Tang, Wanqiu Huang, Yu-Feng Yao

**Affiliations:** 1 Shanghai Institute of Immunology, Shanghai Jiao Tong University School of Medicine, Shanghai 200025, China.; 2 Laboratory of Bacterial Pathogenesis, Department of Microbiology and Immunology, Institutes of Medical Sciences, Shanghai Jiao Tong University School of Medicine, Shanghai 200025, China.; 3 Department of Infectious Diseases, Shanghai Ruijin Hospital, Shanghai 200025, China.; 4 State Key Laboratory of Microbial Metabolism, and School of Life Sciences and Biotechnology, Shanghai Jiao Tong University, Shanghai 200240, China.; 5 Shanghai Key Laboratory of Emergency Prevention, Diagnosis and Treatment of Respiratory Infectious Diseases (20dz2261100), Shanghai 200025, China.

**Keywords:** metabolites, lactic acid bacteria, gut microbiota, immune system

## Abstract

Lactic acid bacteria (LAB) are ubiquitous microorganisms that can colonize the intestine and participate in the physiological metabolism of the host. LAB can produce a variety of metabolites, including organic acids, bacteriocin, amino acids, exopolysaccharides and vitamins. These metabolites are the basis of LAB function and have a profound impact on host health. The intestine is colonized by a large number of gut microorganisms with high species diversity. Metabolites of LAB can keep the balance and stability of gut microbiota through aiding in the maintenance of the intestinal epithelial barrier, resisting to pathogens and regulating immune responses, which further influence the nutrition, metabolism and behavior of the host. In this review, we summarize the metabolites of LAB and their influence on the intestine. We also discuss the underlying regulatory mechanisms and emphasize the link between LAB and the human gut from the perspective of health promotion.

## INTRODUCTION

Lactic acid bacteria (LAB), including *Lactobacillus, Lactococcus, Leuconostoc, Pediococcus, Streptococcus, Aerococcus, Alloiococcus, Carnobacterium, Dolosigranulum, Enterococcus, Oenococcus, Tetragenococcus, Vagococcus*, and *Weissella* [1, 2], are a group of Gram-positive, facultatively anaerobic, non-spore forming, acid tolerating and wildly distributed bacteria which have been widely studied in recent years. LAB can use carbohydrate fermentation to obtain energy and maintain metabolism [3]. LAB proliferate and produce metabolites during fermentation, e.g., organic acids, amino acids, neuroactive molecules, exopolysaccharides, vitamins, and bacteriocins [4]. LAB ferment feed in animal intestines, participate in host metabolism, consume incompletely digested substances, and produce a variety of metabolites [5, 6]. In recent years, LAB research mainly focuses on the probiotic effect and impact on gut microbiota and host health. As members of gut microbiota, LAB exert beneficial characteristics in the intestine by producing metabolites (**Table 1**).

**TABLE 1.** Beneficial characteristics and harmful infections of LAB species in the gut.

**LAB species**	**Beneficial characteristics**	**Ref.**	**Harmful infection types**	**Ref.**
*Lactobacillus acidophilus*	Probiotic characteristics; used for dairy products and biomedical treatment	[111, 112]	Dental abscess/caries; Endocarditis; Bacteremia;	[113, 114]
*Lacticaseibacillus casei*	Probiotic characteristics; used to ferment dairy products; applied in food, biotechnology and medical fields	[115]	Bacteremia; Endocarditis	[114]
*Lactiplantibacillus plantarum*	Probiotic characteristics; ecological and metabolic adaptability; high acid tolerance response; bile salt hydrolysis ability	[116, 117]	Bacteremia; Cholecystitis	[114, 118]
*Lacticaseibacillus rhamnosus*	Probiotic characteristics; acid- and bile-stability; great avidity for human intestinal mucosal cells	[119, 120]	Endocarditis; Bacteremia	[114]
*Lacticaseibacillus paracasei*	Probiotic characteristics; modulate gut microbiota composition and improve gastrointestinal function	[56, 121, 122]	Endocarditis; pancreatic necrosis	[123, 124]
*Lactobacillus johnsonii*	Probiotic characteristics; interact with a great diversity of immune cells; increase the levels of long chain fatty acids	[125, 126]	N/A	
*Lactobacillus delbrueckii*	Probiotic characteristics; bile salt hydrolysis ability	[121, 127]	Urinary tract infection	[128]
*Limosilactobacillus reuteri*	Probiotic characteristics; widely used to prevent and treat numerous gastrointestinal disorders	[111, 129, 130]	Bacteremia	[114]
*Ligilactobacillus salivarius*	Probiotic characteristics; antimicrobial activity; innate immune responses in epithelial cells	[131, 132]	Bacteremia	[133]
*Lactobacillus gasseri*	Probiotic characteristics; used in food industry as supplements; inhibit increase in body weight and adipose tissue weight	[134, 135]	Liver abscesses	[136]
*Ligilactobacillus ruminis*	Probiotic characteristics; modulate inflammatory cytokines and gut microbiota modulation	[137]	N/A	

The gut microbiota contains more than 1500 species distributed in more than 50 phyla, including *Bacteroidetes, Firmicutes, Actinobacteria, Proteobacteria and Verrucomicrobia*. *Firmicutes* and *Bacteroidetes* are the two main phyla of the human gut microbiota [7, 8]. These microbial populations colonize and proliferate in intestinal surfaces, creating a stable system. Once this homeostasis is disrupted by antibiotics, pathogens, or other factors, the gut microbiota imbalance will lead to disorders of multiple physiological functions, such as the endocrine system and digestive system [9–12].

This review highlights the recent advances in LAB and focuses on the metabolites of LAB to summarize their classification and biosynthesis. We explore their modulation of gut microbiota, discuss the limitations of current studies, and potential applications in disease control and health maintenance.

## IMPORTANT METABOLITES OF LAB

### Organic acids

**Figure 1:**
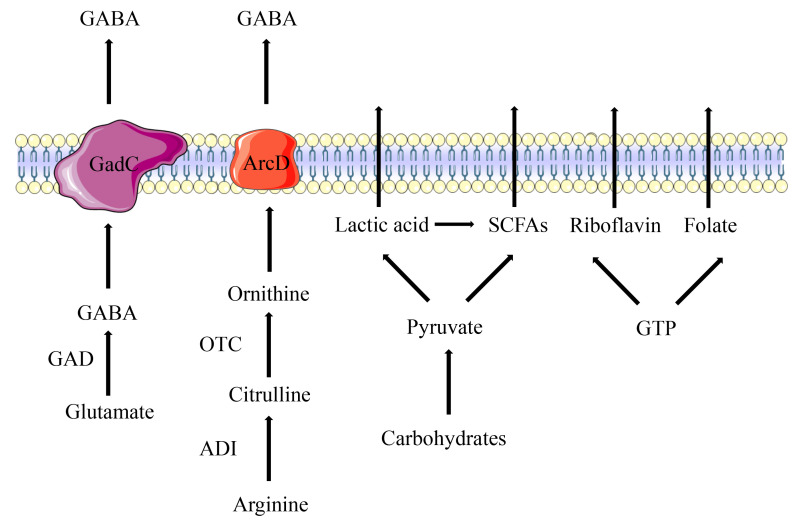
FIGURE 1: Synthesis of amino acids, organic acids and vitamins in LAB. Apart from extracellular polysaccharides and bacteriocin, the synthetic pathways of amino acids, organic acids and vitamins are similar in LAB.

LAB produce organic acids through carbohydrate fermentation in the intestine, which include lactic acid (LA) and short-chain fatty acids (SCFAs). LA is a water-soluble and highly hygroscopic aliphatic acid. LAB are divided into two distinct clades depending on whether they utilize homofermentative or heterofermentative metabolism. Homofermentative LAB convert one molecule of glucose to two molecules of LA, and two molecules of ATP are generated via the aldolase enzyme. Heterofermentative LAB use the alternate pentose monophosphate pathway to convert six-carbon sugars (hexoses) to five-carbon sugars (pentoses), and then produce one molecule of LA and one molecule of ethanol or acetic acid via the phosphoketolase pathway [13, 14]. LA plays an essential role in food fermentation, winemaking, the biopharmaceutical industry and material manufacturing. It is also beneficial for clinical treatment due to its antimicrobial and immunomodulatory properties [15]. SCFAs are volatile saturated fatty acids with less than six carbon atoms in the aliphatic chain, existing in a straight or branched conformation [16]. LAB significantly different in their ability to ferment dietary fibers and sugars to produce different SCFAs. The most abundant SCFAs produced by LAB are acetate, butyrate, and propionate. Acetate can be produced by LAB from pyruvate via acetyl-CoA. In the presence of an electron acceptor, acetyl phosphate is converted to acetate along with the production of additional ATP molecules [17]. Butyrate is produced via either the butyryl-CoA acetate CoA-transferase enzyme or butyryl-CoA. Propionate is mainly formed via the succinate pathway from pyruvate by LAB (**Figure 1**) [18].

### Bacteriocins

Bacteriocins are a group of antibacterial proteins or peptides with broad-spectrum antibacterial activity [19]. They are divided into four classes, including Class I (modified and lanthionine; nisin and antibiotics), Class II (unmodified and linear heat-stable non-lanthionine; <10 kDa), Class III (large heat-labile peptides; >30 kDa), and Class IV (small and circular peptides; <10 kDa) [1, 20]. Different strains secrete their own bacteriocin that are different in their mode of action, molecular weight, genetic origin and biochemical properties [21]. They act on the bacterial cytoplasmic membrane to disrupt membrane vesicles; this property may be desirable when LAB are used to target a specific sensitive pathogen. In food industry, bacteriocins from LAB are biological preservatives in various food products, they are evaluated as Generally Recognized as Safe compounds by the FDA, which means they can be incorporated into foods [1]. The most widely used bacteriocin is nisin, which are produced by *Lactococcus lactis* strains. Nisin is a well-known food preservative in commercial development and production. It has wide a antimicrobial spectrum and decomposition capabilities mediated by digestive enzymes [22].

In the last few decades, many bacteriocins have been identified and applied. *Leuconostoc lactis* SM2 was found to produce Leucocin, with maximum antimicrobial activity against target bacterial strains [23]. *Lactiplantibacillus plantarum* NWAFU-BIO-BS29 was found to produce a novel potential bacteriocin of Plantaricin Bio-LP1, which is a bio-preservative with a high ability to inhibit and kill pathogens [24]. Bacteriocin KCA produced by *Carnobacterium maltaromaticum* KCA018 showed narrow spectrum antimicrobial activity, effectively inhibiting Gram-positive bacteria, and also has potential as a bio-preservative [25].

### Amino acids

LAB have four amino acid metabolic pathways, including the arginine deiminase (ADI) pathway, the glutamate decarboxylase (GAD) system, the branched chain amino acid (BCAA) metabolism, and the aromatic amino acid metabolism [26]. In the ADI pathway, arginine is converted into citrulline and ammonia by arginine deiminase (ArcA), and then citrulline is converted into ornithine and carbamoyl phosphate by ornithine transcarbamylase (**Figure 1**). The arginine/ornithine antiporter is responsible for transporting external arginine into the cells and expelling ornithine outside the cells [27]. The ADI pathway is a bacterial trait found most abundantly among *Lactobacillales*. Amino acids involved in these pathways have been reported to provide benefits for human health. For example, citrulline is a non-essential amino acid that has been used for treatment of intestinal inflammation [28]. Ornithine is an essential amino acid that has several important physiological functions such as anti-obesity activity, stimulation of hormone growth and promotion of muscle growth [29].

Among the amino acid metabolites of LAB, there is a neuroinhibitory molecule called γ-Aminobutyric Acid (GABA), a non-protein amino acid widely found in animals, plants and microbiota. GABA is a major inhibitory neurotransmitter in the mammalian central nervous system [30]. Due to its benefit in reducing the activity of neurons, preventing nerve cells from overheating and decreasing blood pressure, GABA is wildly used in treatment of psychiatric disorders such as epilepsy, convulsion and Parkinson's disease [31]. In LAB, GABA is irreversibly activated by pyridoxal 5′-phosphate (PLP)-dependent GAD under anaerobic conditions to decarboxylate L-glutamate, and the whole process is accompanied by the consumption of an H^+^ (**Figure 1**). After intracellular synthesis, the Glu/GABA antiporter GadC is responsible for transferring intracellular synthesized GABA to the outside and transporting extracellular glutamate inside [32]. GABA-producing LAB, such as *Lactiplantibacillus paraplantarum* Y7 [32] *L. paraplantarum* FBT215 [33], *Levilactobacillus brevis* CGMCC1.5954 [34], *Lacticaseibacillus rhamnosus* 21D-B, *Lacticaseibacillus paracasei* 15C, and *Streptococcus thermophilus* 84C [35], have been isolated from various fermented foods and have been constantly optimized.

### Exopolysaccharides

Exopolysaccharides (EPSs) are long-chain polysaccharides consisting of branched, repeating units of sugars or sugar derivatives synthesized by microorganisms [36, 37]. In LAB, EPSs typically exist in the cell surface or slime and can be secreted. They are classified into two types: homopolysaccharides (HoPS) and heteropolysaccharides (HePS). HoPS are polymerized by only one monosaccharide, such as dextran or fructan [38]. The majority of HoPS-producing LAB are *Leuconostoc mesenteroides* [39], *Leuconostoc citreum* [40], and *S. thermophilus* [41] isolated from animal, vegetables and fermented food. Compared with HoPS, HePS are composed of repeating units of different monosaccharides, mainly including glucose, galactose or rhamnose in different proportions. Some HePS also contain N-acetyl-D-glucosamine, N-acetyl-galactosamine, uronic acid or some non-carbohydrate substituents, such as pyruvic acid, acetate, phosphate or succinate [42]. The most prominent HePS-producing LAB are plant-derived *Lactobacillus paracasei* [43], *L. lactis,* and *Lactococcus cremoris* [44] and are frequently isolated from fermented products and human feces. EPSs from LAB show anti-cancer, anti-inflammatory and antioxidant activities and have been applied in foods, biomedical and cosmetic industries [36].

### Vitamins

Although LAB are considered as auxotrophic bacteria for several vitamins, they are able to synthesize B group vitamins such as riboflavin, folate, thiamine and cobalamin [45]. Riboflavin is synthesized in a complex pathway, involving seven enzymatic stages from guanosine triphosphate (GTP), d-ribulose-5-phosphate precursors, purine metabolism and pentose phosphate pathway [46]. Riboflavin is the precursor of flavin mononucleotide and flavin adenine dinucleotide, these two coenzymes assist in the metabolism of carbohydrates, amino acids and energy production [47] *Limosilactobacillus reuteri* AMBV339 represents a promising candidate to provide riboflavin fortification of plant-based and dairy foods [48]. Riboflavin produced by *L. plantarum* CRL2130 significantly decreased the release of interleukin (IL)-6 and the formation of reactive oxygen [47]. Folate participates in numerous vital biological reactions, including DNA synthesis and methylation. It cannot be synthesized by humans and must be obtained exogenously (**Figure 1**) [49]. The synthesis of folate in LAB comprises 17 enzymatic reactions. Meanwhile, the ability to produce folates is strain-specific and is influenced by growth conditionsm[46]. *Lactobacillus casei, L. plantarum, L. paracasei* subsp*. paracasei, Lactobacillus rhamnosus, S. thermophilus, L. lactis* subsp*. lactis, Enterococcus faecium*, and *Enterococcus lactis* are able to synthetize folates in the medium [50].

## EFFECTS OF LAB ON THE GUT MICROBIOTA

### LAB promote stability of the gut microbiota

The gut microbiota is complex, and its stability is related to the health of the host. Generally, the species, quantity, and composition of microorganisms in the gut microbiota tend to be stable in a healthy state [51]. LAB can avoid a sharp increase or decrease in the number of a certain flora in the intestine and reduce the number of pathogenic bacteria, and thus maintain homeostasis. Once this balance is disturbed, it will result in intestinal disease and affect the health of the host. In this case, LAB can restore the balance of gut microbiota by changing its composition and promoting its stability [52].

The species, quantity and proportion of microorganisms in the intestine constitute the unique structure of gut microbiota in the animal intestine. In healthy hosts, the structure of gut microbiota is in a dynamic balance where the addition of LAB has little impact on the structure and composition of the gut microbiota [53]. However, the number of pathogenic bacteria increases dramatically and the proportion of probiotics is small in patients with intestinal diseases, in this case, LAB play a leading role in shaping the gut microbiota into a certain state. It alleviates the imbalance of the gut microbiota caused by diseases through upregulating the content of beneficial bacteria and reducing pathogenic bacteria. A study showed that *Lactobacillus reuteri* CCFM8631 can increase the relative abundance of faecal Deferribacteres, *Lachnospiraceae* NK4A136 group, while *Lactobacillus* and *Dubosiella* decrease the relative abundance of *Erysipelatoclostridium* and *Romboutsia* in mice with cardiovascular disease [54]. In high-fat diet mice, the diversity of the gut microbiota and its composition were significantly changed after treatment with probiotics that contained LAB, the number of *Lactobacillales, Clostridiales*, and *Bifidobacteriales* was over-represented while the phylum *Verrucomicrobia* was absent and the number of Proteobacteria was significantly lower in the probiotic-treated group compared to placebo-treated high-fat diet fed mice [55]. In aged mice, next-generation sequencing results revealed that the probiotic *L. paracasei* PS23 treatment enriched *Lactobacillus* and *Candidatus* while the abundance of *Lachnospiraceae*_UCG_001 was under-represented [56]. The content of probiotics such as *Bifidobacteriales* or *Ruminococcus* was effectively over-represented in ulcerative colitis mice after the probiotic treatment, and the imbalance of the gut microbiota was further improved [57]. LAB also play an important role in the alteration of microbial diversity of the gut microbiota in colorectal cancer (CRC) pathogenesis. LAB have been used to manufacture probiotic preparations to modulate gut microbiota so as to reduce the risk of CRC. One clinical study showed that LAB changed the gut microbiota composition by increasing the abundance of beneficial bacteria such as *Eubacterium, Peptostreptococcus*, and *Bifidobacterium*, while the abundance of potentially harmful bacteria such as *Fusobacterium, Porphyromonas*, and *Enterococcus* was under-represented. In this way, LAB can improve the ability of the gut microbiota to resist CRC [58]. Another study focused on the gut microbiota in crew of ships that make long voyages and observed that the stressful ship voyage significantly decreased the diversity of gut microbiota at the end of the voyage, and this change can be recovered through probiotic preparations enriched with LAB [59, 60].

Besides increasing the content of probiotics and reducing the content of pathogenic bacteria, LAB are also able to change the abundance of related bacteria in the gut microbiota to alleviate specific diseases. For example, *Bacteroidetes* and *Firmicutes* are two dominant populations in the gut, and the ratio between *Bacteroidetes* and *Firmicutes* (F:B) is a distinct feature of the gut microbiota, which is significantly reduced in obese mice. The F:B ratio is widely used as a sign of gut dysbiosis in hypertension. Some studies mentioned that in spontaneously hypertensive rats, the F:B ratio was significantly higher than in normal rats. However, after treatment with probiotic yogurt which contains *Lactobacillus bulgaricus*, the F:B ratio returned to a lower level. In addition, the phylum level of the gut microbiota in the probiotic yogurt treatment group was similar to that in the normal group, while the composition of the gut microbiota communities in hypertension was restored after probiotic yogurt treatment [61]. Another study found that the gut microbial composition completely shifted after ovariectomy in rats, the proportions of *Lachnospiraceae* and *Ruminococcaceae* were over-represented and the proportion of *Muribaculaceae* was under-represented. *Lactobacillus intestinalis* YT2 treatment could restore the relative abundance of these three most impacted microorganisms and counteract the ovariectomy-induced dysbiosis [62]. Furthermore, researchers found that *Bifidobacterium* and *Atopobium* clusters of the *Actinobacteria* phylum were maintained at higher counts after LAB treatment compared with the untreated group in patients with depressive symptoms, indicating that LAB are beneficial to alleviate depressive symptoms [63].

### Metabolites participate in the modulation of the gut microbiota

**Figure 2:**
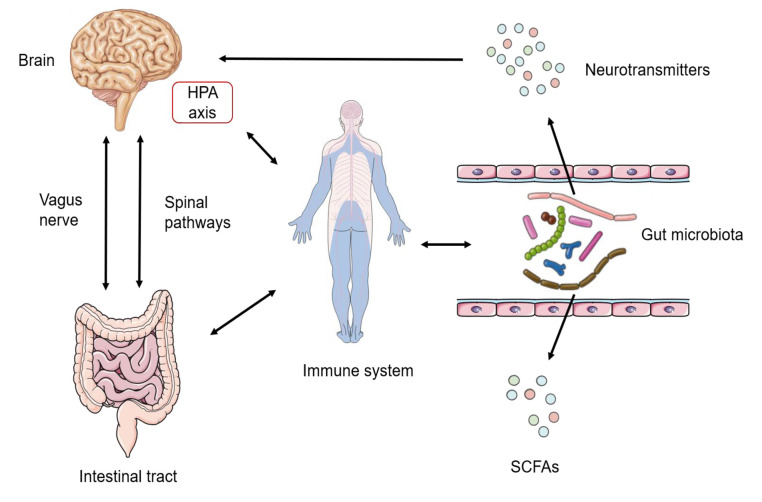
FIGURE 2: Routes of communication in the gut-brain-axis (GBA). The Gut microbiota is one of the key regulators of gut-brain function. Communication within the occurs through these three ways: the immune pathway, the neuroendocrine pathway (neurotransmitters), and the vagus pathway, involving microbial metabolites such as SCFAs. LAB maintain homeostasis and affect host behaviors via the microbiota–gut–brain axis. HPA, hypothalamic–pituitary–adrenal axis, is central to homeostasis, stress responses, energy metabolism, and neuropsychiatric function.

Homoeostasis of the gut microbiota is orchestrated by multiple host regulators such as diet, immune system and nervous system. Diet is the most important host-dependent regulator, which has been widely recognized as the critical determinant of differences of the intestinal flora among individuals. Besides, the immune system also plays a crucial role in regulating the gut microbiota: the change of sensors of the innate immune system, defects in anti-microbial peptide secretion by Paneth cells or endoplasmic reticulum stress response are all essential regulators of gut microbial ecology [64, 65]. The nervous system is another crucial factor regulating the gut microbiota. The gut–brain axis (GBA) has been proved to provide a channel for the central nervous system (CNS) and the gut microbiota (**Figure 2**). Signals from the CNS are directly passed to the enteric nervous system (ENS), and are involved in the control of the gut microbiota through neuronal circuits [66]. Bidirectionally, the gut microbiota can communicate to the brain through several routes: bacterial metabolites (e.g., SCFAs, folate and EPS), production of neurotransmitters (e.g., GABA and 5-hydroxytryptamine (5-HT)), enteroendocrine cell activation (e.g., glucagon-like peptide 1), immunomodulation (e.g., IL-6 and tumor necrosis factor-alpha (TNF-α)), and stimulation of the ENS and the vagal nerve [67–69]. Apart from the factors mentioned above, stability of the intestinal environment, invasion of pathogenic bacteria, drug treatment, emotional changes and other factors are involved in the complex regulatory network of the intestinal flora.

The effect of LAB on gut microbiota is significant, which makes LAB a widely used probiotics to maintain human and animal health. These advantages are derived from the physical and chemical properties or surface components and metabolites. To date, a few studies have been conducted to specifically investigate the effects of LAB metabolites on the gut microbiota. Nonetheless, among the many metabolites of LAB, some are nutritional substrates for intestinal bacteria or cells, some are neurotransmitters involved in neural regulation, and some are antibacterial substances that inhibit pathogenic bacteria. These properties indicate that the metabolites of LAB have an important impact on the regulation of the gut microbiota.

## MECHANISMS OF METABOLITS MODULATING THE GUT MICROBIOTA

### Metabolites of LAB protect the intestinal epithelial barrier

Intestinal epithelial cells (IECs) are important structural components of the intestine, and are closely related to the intestinal functions: nutrition, absorption, and mucus secretion. Epithelial cells include intestinal cells, colon cells, lymphocytes, and goblet cells; they constitute the single layer of epithelial cells of the intestine. Multiple single-layer cells form the intestinal epithelial cell barrier together, which is not only the barrier separating the external and internal environment of the intestine, but also connect the gut microbiota and the host. SCFAs released by the gut microbiota maintain the normal function of the intestinal epithelial barrier. Butyrate is the main SCFA in the intestine, it can be quickly absorbed by intestinal epithelial cells and used as an energy substrate. LAB can benefit the growth of intestinal epithelial cells by releasing butyric acid, and can also promote the synthesis of trefoil factor that is related to mucosal repair, thereby further boosting intestinal epithelial barrier function [70]. Moreover, studies have shown that the expression of tight junction proteins (TJp) could be promoted by microbial-derived butyrate, which could also stimulate goblet cells to secrete mucin [70]. Butyrate is a histone deacetylase inhibitor and has been reported to bind to specific G-protein-coupled receptors. In IECs inflammatory responses induced by exogenous lipopolysaccharide from pathogens, sodium butyrate activates G-protein-coupled receptors 43 to protect them against the response [71]. Besides butyrate, acetate and propionate can increase the intestinal barrier integrity by enhancing (TJp functions, thus the passage of antigens through the paracellular space will be prevented [72]. SCFAs can also neutralize the toxicity of some toxins secreted by pathogens. Scientists designed a probiotic preparation containing three LAB species to treat *Clostridioides difficile* infection (CDI), this preparation had a strong inhibitory effect on the growth of nosocomial *C. difficile* strains by producing SCFAs and other organic acids that neutralize and restrain their toxicity during fermentation, preventing *C. difficile* strains from harming IECs and the gut microbiota [73]. While neutralizing toxins, butyrate stabilizes hypoxia-inducible factor 1α (HIF-1α) in IECs and can protect them from damage caused by *C. difficile* toxins, mitigating local inflammatory response and systemic consequences of the infection [74]. Butyrate is an inhibitor of the C-X-C motif chemokine ligand 10 (CXCL10) release and a transducer and activator of the transcription 1 (STAT1) signaling cascade. The activation of these cascades and release of pro-inflammatory CXCL10 will accelerate Th-1 type inflammatory responses. Butyrate reduces the release of CXCL10 and STAT1 signaling cascade via histone deacetylase inhibiting, thereby reducing inflammation and maintaining intestinal barrier integrity [75].

### Metabolites of LAB inhibit intestinal colonization by pathogens

Pathogens are the primary factor threatening intestinal homeostasis. They disturb the gut microbiota through their own toxicity and nutritional competition or directly invade the host epithelial cells. Pathogens in the intestinal tract adhere to epithelial cells and absorb nutrients from the intestine to survive and proliferate. Metabolites of LAB can inhibit the adhesion of pathogens or directly kill them to restrain the intestinal colonization of pathogens.

Adhesion to human IECs is considered as one of the most important characteristics that allow LAB to achieve their probiotic attributes [76]. The adhesion of LAB is mostly dependent on adhesin recognizing the extracellular matrix of IECs. LAB adhesins are categorized into protein adhesins and glycan-mediated adhesins and include proteins, peptides, glycolipids, sugars, and other multifunctional metabolic molecules with multiple structures. Adhesins bind to receptors in the extracellular matrix of IECs and intestinal mucus such as collagens, proteoglycans, laminins, hyaluronans, or elastin. These adhesins and receptors assemble into diverse adhesive structures on the intestinal epithelium, which plays a foremost role in LAB residence in the intestine [77]. When LAB colonize the intestinal epithelium successfully, they occupy adhesion sites and thereby block pathogenic bacteria from adhering to the same part, thus preventing the diffusion and translocation of pathogenic bacteria. For example, binding of S-layer proteins from *Lactobacillus acidophilus* to the cellular receptor dendritic cell (DC)-specific intercellular adhesion molecule-3-Grabbing Non-integrin (DC-SIGN) is a common adhesion mechanism. Researchers used S-layer proteins to bind to DC-SIGN-expressing cells and then checked the infection rate of bacteria. The results showed that after treatment with S-layer proteins, the infection rate was diminished by up to 79% for both Gram-negative and mycobacterial models and the bacteria viability was reduced [78].

Among the metabolites of LAB, bacteriocins can kill pathogens and inhibit growth of closely related bacterial strains [19]. Some studies have found that the bacteriocins released by LAB could exert the antibacterial function through acting on the cell membrane, cell wall, or cell cycle. They form holes in the cell membrane of sensitive pathogenic bacteria, increase the permeability of cell membranes, disrupt the proton gradient of the target cell membrane, destroy the integrity of the cell membrane, thereby causing the release of cellular content, leading to the death of pathogenic bacteria [79, 80]. Studies have found that nisin and garvicin ML, two bacteriocins from LAB with broad-spectrum antimicrobial activities, have obvious inhibitory and physicochemical properties on several intestinal bacterial groups, without having a great impact on the gut microbiota particularly in the healthy intestine [81]. The chemically synthesized bacteriocins GarAG1 and GarAG2 from *Lactococcus garvieae* presented inhibitory activity against pathogenic *L. garvieae* strains, with AG2 also being active against *Listeria monocytogenes, Listeria ivanovii* and *E. faecalis* [82]. In constipated mice, bacteriocin-producing *Pediococcus acidilactici* strains significantly reduced the levels of *Bacteroides* and genera from *Enterobacteriaceae* and alleviate constipation-related symptoms [83]. Some studies found that the circular peptide gassericin A from *Lactobacillus gasseri* LA39 and *Lactobacillus frumenti*, a bacteriocin which can bind to Keratin 19 (KRT19) on the plasma membrane of IECs, was essential for enhancement of fluid absorption and decreased secretion that increases resistance to diarrhea in early-weaned piglets [84]. Studies have shown that bacteriocins not only act on attacking pathogens but also act as colonizing peptides for certain intestinal microorganisms, promoting these bacteria to acquire a competitive advantage over other strains and occupying established niches in the intestines [70].

Under aerobic conditions, LAB can secrete another antibacterial substance, hydrogen peroxide (H_2_O_2_), produced through the activity of nicotinamide adenine dinucleotide (NADH) oxidase, superoxide dismutase and other enzymes. Hydrogen peroxide can reduce the virulence of pathogenic bacteria, intervene with the invasion of epithelial cells by pathogenic bacteria and kill pathogenic bacteria that spread in IECs directly. Researches showed that the combination of hydrogen peroxide and lactic acid could effectively kill pathogenic bacteria as well as fungi by altering the permeability of the membrane [85].

### LAB metabolites regulate the immune system to modulate the gut microbiota

The intestine is an important part of the immune system, including a variety of immune organs and immune cells. The gut microbiota is strongly impacted by the immune system. As beneficial bacteria in the intestine, LAB often regulate the intestinal immune system by stimulating and promoting the function of the immune system or affecting the growth and enrichment of immune cells via different metabolites, thereby maintaining the balance and stability of the gut microbiota.

The mucosal immune system includes many proteins such as mucins and TJps and is the most crucial non-specific immune system in the intestine where the encounters with pathogens occur. Butyrate can reinforce this barrier by increasing the number of goblet cells which secrete mucins and build the mucus layer. It facilitates M2 macrophage polarization with the elevated expressions of CD206 and arginase-1 (Arg1), and then increases mucin production and the proportion of mucin-secreting goblet cells in the colon crypt in a macrophage-dependent manner by using clodronate liposomes [6]. Butyrate can also stimulate pathways involved in the production of defensins, which are cationic antimicrobial peptides produced by small intestinal recess cells and Paneth cells in IECs, strengthening the integrity of the intestinal mucosa and protecting the intestine against pathogens with the help of leucine [86]. Furthermore, LAB can also use ornithine to influence the proliferation of mucosal cells and the production of mucin via gut immune cells. Ornithine increases the level of aryl hydrocarbon receptor ligand L-kynurenine produced from tryptophan metabolism in IECs, which in turn increases ILC3 cells [29]. Secretory IgA is a multifunctional protein in mucosal-associated immunity which can bind antigens to avoid pathogen infections. MM89-EPS, an EPS isolated from *L. plantarum* MM89, was reported to increase intestinal IgA levels in mice [87]. In order to cope with pathogenic infections effectively and properly, the intestinal immune system needs to remain in a high-alert-state to respond to signals such as cytokines in time. However, excessive response to this cytokine will cause inflammation, which requires that the immune cell response is controlled at an appropriate level. LAB can control the production of cytokines such as IL-6 or IL-12 and promote the development of IECs, macrophages and T cells that can produce cytokines [88]. Studies have found that SCFAs significantly modified the expression of IL-1β and IL-6 and inhibited intestinal recruitment of neutrophils to keep the inflammation at proper levels in zebrafish [89].

The gut microbiota mainly takes part in immune regulation through the Toll-like receptor (TLR)-dependent nuclear factor-kappa B (NF-κB) pathway. LAB can modulate the NF-κB signal directly or indirectly through their active biological molecules at different sites [90, 91]. A novel EPS (EPS-3A) from *S. thermophilus* (ZJUIDS-2-01) can activate macrophages through MAPKs and NF-κB signaling pathway [42]. TLRs are important pattern recognition receptors, which are expressed mainly by macrophages, DCs, B cells and epithelial cells [92]. TLRs can recognize pathogen-associated molecular patterns and initiate the response of DCs, which leads to the production of cytokines and upregulation or downregulation of cell surface molecules whose signals critically influence further induction of both innate and adaptive immunity [93].

As for adaptive immunity, gut-derived or exogenous metabolites of LAB both have impact on T cells including T-helper 1 (Th1), T-helper 2 (Th2), Treg and TH17 [94, 95]. These functions depend on SCFAs that can directly or indirectly regulate T-cell differentiation into functionally specialized cells especially in Th1 and Th17 when T-cells undergo antigen priming by antigen presenting cells in the presence of SCFAs [96]. Bulgaricus OLL1073R-1 (EPS-R1) from *Lactobacillus delbrueckii* has been reported to have anti-tumor activity, as it can help with infiltrating CCR6+ CD8+ T cells and producing IFNγ accompanied by a substantial immune response gene expression signature maintaining T-cell functions in mice [97]. In B cells, SCFAs increase acetyl-CoA and decrease AMP levels and AMP activated protein kinase activity, regulate metabolic sensors to increase oxidative phosphorylation, glycolysis, and fatty acid synthesis, so as to support antibody production [98]. LAB can also produce different vitamins to resist intestinal inflammation and regulate the immune system. It has been confirmed by researches that riboflavin-producing and folate-producing LAB can significantly decrease IL-6 and TNF-α levels to attenuate recurrent inflammation [99].

### LAB metabolites affect host behavior

The relationship between the gut microbiota and host behavior has been a research hotspot in recent years. One of the major areas of investigation is the role of the microbiome in emotional experience. Research efforts have expanded to study the effects of psychobiotics, specific probiotic strains that modulate neurotransmitters, neurotrophic factors and behaviors [100]. Studies show that the gut microbiota composition is commonly associated with mental state andpeople with mental disorders tend to have higher levels of LA-producing bacteria and higher levels of bacteria associated with glutamate and GABA metabolism compared to healthy people [101]. LAB have been shown to produce mammalian neurotransmitters to alter neurotransmitter levels. In immune pathways, LAB are capable of producing histamine to participate in gut immunity and exert anti-inflammatory effects by modulating interleukin-18 production in the gut [102]. In the vagus pathway, GABA and its receptors are widely distributed in mammalian hosts and are the main inhibitory neurotransmitters in the CNS. GABA produced by LAB mainly plays a role through the vagus nerve pathway. Studies have shown that low GABA levels or dysfunction can cause depression, while *Lactobacillus murine* and *L. reuteri* can restore GABA levels to alleviate depressive symptoms in mice [103]. Studies also found that L-dopa produced by *E. faecalis* or *E. faecium* circulates into the brain and is converted to dopamine, a predominant neurotransmitter, via the phenylalanine-tyrosine-dopa-dopamine pathway [104]. *L. plantarum* PS128 (PS128) ameliorated abnormal behaviors and modulated neurotransmissions in dopaminergic pathways in rodent models, and significantly increased methionine biosynthesis-related microbial modules [105]. In addition, *L. rhamnosus* GG supernatant upregulated the expression of 5-HT receptor 4. *Ligilactobacillus salivarius* Li01 also promoted the expression of 5-HT receptor 4 and decreased the levels of 5-HT in serum [106, 107]. These observations suggested roles of LAB metabolites in modulating host behavior.

## CONCLUSION AND FUTURE PERSPECTIVES

**Figure 3:**
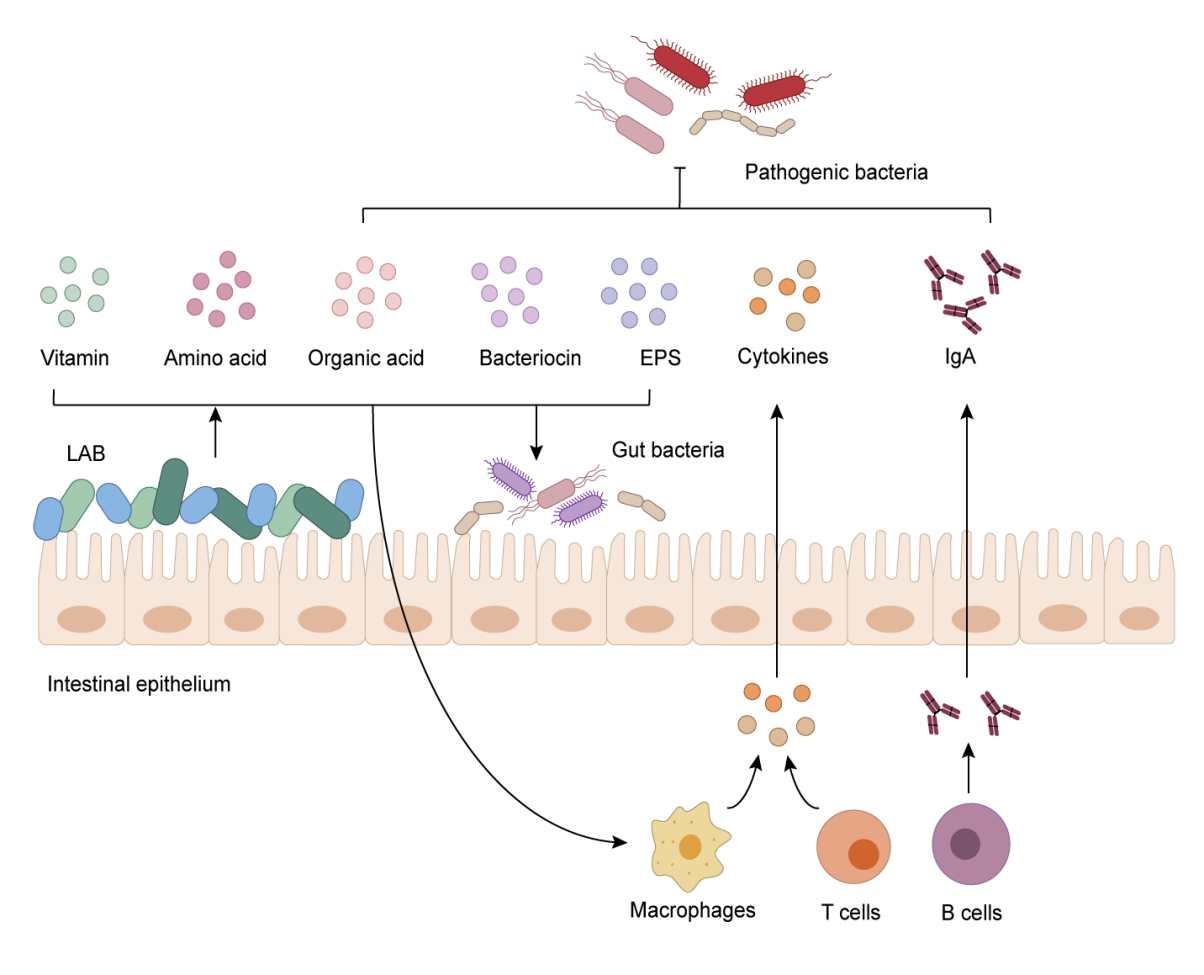
FIGURE 3: The regulatory mechanism of the gut microbiota by LAB. LAB inhibit invasion and colonization of pathogens through occupying adhesion sites and producing metabolites like organic acid, bacteriocin, and EPS. LAB also activate the host innate immune system and promote the secretion of cytokines and IgA, which strengthens the integrity of intestinal mucosa and protects the intestine against pathogens.

LAB produce a variety of metabolites, including organic acids, bacteriocin, amino acids, EPS and vitamins. These metabolites are the basis of the regulation function of LAB and have a profound impact on host health (**Figure 3**). Plenty of studies have elucidated the significant impact of LAB on host health. The impact is mainly based on regulatory effects of LAB on the gut microbiota. LAB can directly or indirectly ensure the stability and homeostasis of the gut microbiota through itself and its metabolites, which in turn act on intestinal cells and affect host health. This regulation is the basis for the intestinal tract to maintain health and resist pathogens. Many studies have focused on the role of LAB in regulating the gut microbiota, but few studies have defined the decisive components of LAB that play a critical function, such as its surface compound or metabolites. On the one hand, many clinical cases reported infections caused by LAB, showing that LAB are not harmless microorganisms (**Table 1**). On the other hand, not all metabolites are beneficial. When preparing LAB preparations, we must consider the impact of the host's original microbiota. LAB metabolites, such as LA, butyric acid, GABA, and ornithine, which have been industrialized and are being studied, are relatively common and can be synthesized by the intestinal flora, they can be detected in the intestine. When it comes to the treatment of diseases with specific components of LAB, drug efficiency and side effects need to be significantly improved, which will be a challenge for research and commercial production of LAB.

With regard to LAB metabolites, many questions remain unanswered currently. Firstly, some metabolites are not only produced by microorganisms but by hosts. To distinguish the role and effect of metabolites produced by hosts and microorganisms will be helpful to study the actual role of LAB metabolites. Secondly, in addition to the several mechanisms mentioned above, LAB might also modulate the gut microbiota in other potential ways. LA has been found to be involved in the post-translational modification of histones and consequently impacts gene expression [108], and propionate and butyrate can affect bacterial virulence through acetylation [109]. It remains to be elucidated whether metabolites regulate the post-translational modification of some regulatory factors in the gut microbiota. Additionally, whether different metabolites are synergistic in regulating the gut microbiota still needs to be explored. With the development of the Next Generation Probiotics (NGP), metabolites are becoming important indicators for the assessment of benefits and safety of probiotics. NGP are live microorganisms identified on the basis of comparative microbiota analyses that, when administered in adequate amounts, confer a health benefit to the host. Different from traditional probiotic strains, NGPs are identified using next generation sequencing techniques and bioinformatics tools [110]. Therefore, the systematic integration of microorganisms and their metabolites has a vital contribution to study the complex interactions between microorganisms-microorganisms and microorganisms-hosts.

## Abbreviations:

ADI: – arginine deiminase pathway;
CNS: – central nervous system;
CRC: – colorectal cancer;
DC: – dendritic cell;
ENS: – enteric nervous system;
EPS: – exopolysaccharide;
GABA: – γ-aminobutyric acid;
GAD: – glutamate decarboxylase system;
HePS: – heteropolysaccharide;
HoPs: – homopolysaccharide;
IEC: – intestinal epithelial cell;
IL: – interleukin;
LA: – lactic acid;
LAB: – lactic acid bacteria;
SCFA: – short-chain fatty acid;
TJp: – tight junction protein;
TLR: – toll-like receptor.
